# Non-ketogenic combination of nutritional strategies provides robust protection against seizures

**DOI:** 10.1038/s41598-017-05542-3

**Published:** 2017-07-14

**Authors:** Glenn Dallérac, Julien Moulard, Jean-François Benoist, Stefan Rouach, Stéphane Auvin, Angèle Guilbot, Loïc Lenoir, Nathalie Rouach

**Affiliations:** 1 Neuroglial Interactions in Cerebral Physiopathology, Center for Interdisciplinary Research in Biology, Collège de France, CNRS UMR 7241, INSERM U1050, Labex Memolife, PSL Research University, Paris, 75005 France; 2 AP-HP, Hôpital Robert Debré, Service de Neurologie Pédiatrique, Paris, France; 3 PILEJE Laboratoire, 37 quai de Grenelle, 75015 Paris, France

## Abstract

Epilepsy is a neurological condition that affects 1% of the world population. Conventional treatments of epilepsy use drugs targeting neuronal excitability, inhibitory or excitatory transmission. Yet, one third of patients presents an intractable form of epilepsy and fails to respond to pharmacological anti-epileptic strategies. The ketogenic diet is a well-established non-pharmacological treatment that has been proven to be effective in reducing seizure frequency in the pharmaco-resistant patients. This dietary solution is however extremely restrictive and can be associated with complications caused by the high [fat]:[carbohydrate + protein] ratio. Recent advances suggest that the traditional 4:1 ratio of the ketogenic diet is not a requisite for its therapeutic effect. We show here that combining nutritional strategies targeting specific amino-acids, carbohydrates and fatty acids with a low [fat]:[proteins + carbohydrates] ratio also reduces excitatory drive and protects against seizures to the same extent as the ketogenic diet. Similarly, the morphological and molecular correlates of temporal lobe seizures were reduced in animals fed with the combined diet. These results provide evidence that low-fat dietary strategies more palatable than the ketogenic diet could be useful in epilepsy.

## Introduction

Epilepsy is a neurological condition that affects ~65 million people worldwide. The development of antiepileptic drugs in the second half of the 20^th^ century has provided an efficient means of controlling seizures for a large proportion of epileptic patients. However, approximately 30% of patients display intractable forms that do not respond to antagonists of sodium or calcium channels, glutamate receptors, nor to enhancers of gamma-aminobutyric acid (GABA) mediated inhibition^1^. In such cases, a common solution is the ketogenic diet (KD), which was developed in the early 1920s and designed to mimic the metabolic mode of fasting. KD causes body fat to be converted into ketone bodies, which can then be used as a source of energy. To achieve this, the diet must comply to 4 portions of lipids for 1 portion of carbohydrates + proteins, which typically leads to 90% of the energy being provided by fat, 7–8% by proteins and only 2–3% by carbohydrates^2^. Despite the effectiveness of the KD, which reduces seizures by at least 50% in approximately half of the enrolled patients, this dietary solution is very demanding and difficult to follow, such that it is almost exclusively applied to the treatment of children experiencing intractable epilepsy^2^. In these instances, KD is indeed a valuable way of controlling seizures during critical periods of development. However, KD is associated with systemic complications, including growth retardation, nephrolithiasis, and hyperlipidaemia^3^.

Although the mechanisms by which KD protects against seizures remain poorly understood, it has been proposed that reducing the activity of the glycolysis enzyme lactate dehydrogenase (LDH) would be sufficient to reduce seizure occurrence^4^. This implies that it is not ketone bodies but rather the drastic reduction in glucose, which shuts down the LDH metabolic pathway, that is protective. Consistent with this, the relationship between seizure control and serum levels of β-hydroxybutyrate (βHB) and acetoacetate (ACA), the two main ketones produced in KD, is far from clear^5^. Most interestingly, several other dietary strategies have been explored to modify or replace KD. One of the first modifications of the KD consisted in replacing part of the lipids typically provided as long-chain fatty acids with medium-chain fatty acids^6^. These indeed present a higher ketogenic potential as they are absorbed more effectively and carried directly to the liver by the portal blood. The original version of this diet contained 60% of medium-chain triglycerides, a proportion ensuring ketosis. However, such high amounts of medium-chain triglycerides were found to often provoke abdominal pain and gastrointestinal disorders^7^, so that it is now advised to use medium- and long-chain triglycerides in equal measures^8^. The use of polyunsaturated fatty acids may also be beneficial against seizures, as shown for omega-3 fatty acids both in animal models^9, 10^ and in humans^11, 12^, although their efficacy is not obvious in patients^13, 14^. An alternative approach has been to target carbohydrates, not through restriction but by making sure they do not elevate blood sugar levels. Such low glycaemic index treatment has provided good protection against seizures in the small number of clinical trials reported^15^, despite weaker and more variable levels of ketosis than the KD. Accordingly, the efficacy of the low glycaemic index treatment was found not to correlate with the degree of ketosis but rather with the decrease in blood glucose^16^. Also supporting this approach, glycolytic inhibition with 2-deoxyglucose administration has proved effective in reducing seizure occurrence in epilepsy models^17, 18^. Finally, interesting data suggest that the proportion of branched-chained (BCAA) *vs* aromatic amino acids (AAA) in the blood can influence excitability of central neuronal networks^19, 20^. The BCAAs leucine, isoleucine and valine would indeed favour ketosis and GABA synthesis while reducing glutamate levels^19, 21^. Thus, a higher BCAA/AAA blood ratio has been proposed to decrease brain excitability, thereby favouring seizure control^20^. Accordingly, L- and D-Leucine have been found to exert a potent anti-seizure effect^22^.

In the current study, we sought to design a healthy and more palatable diet with effective anti-epileptic properties by combining different nutritional strategies. This new combined diet (CD) comprises medium-chain fatty acids, polyunsaturated fatty acid, low glycaemic index carbohydrates, and a high BCAA/AAA ratio. We first ascertained whether CD could induce the downregulation of excitatory drives associated with the traditional KD^4, 23^. Antiepileptic efficacy of the CD was then tested on susceptibility to seizures and on the chronic kainate (KA) mouse model of epilepsy. Cytological and molecular correlates of seizure occurrence in KA mice were also compared between diets.

## Results

### Design of a diet combining nutritional strategies to achieve a low [fat]:[proteins + carbohydrates] ratio

**Figure 1:**
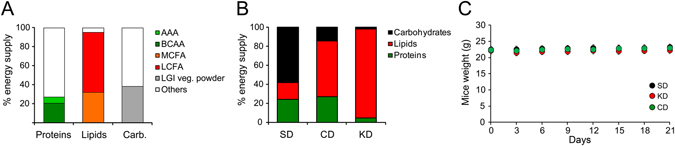
Novel diet combining nutritional strategies reducing the ratio of [fat]:[proteins + carbohydrates]. (**A**) combined diet composition of the total caloric supply. (**B**) proportion of macronutrients caloric supply for SD, CD, and KD. (**C**) monitoring mice weight every third day upon 3 weeks of dietary treatment revealed no change in body mass.

We aimed at composing an antiepileptic diet with a ratio of [fat]:[proteins + carbohydrates] which moderately deviates from a standard diet (SD), by combining macronutrients reported to reduce neuronal excitation. Hence, the fatty acid content of our combined diet was enriched in both medium-chain and polyunsaturated fatty acids. Nutritional analyses showed that they respectively provided 31.9% and 17.2% of the lipid energy content of the CD. In addition, 38.5% of the carbohydrate caloric supply was provided by a powder of low glycaemic index vegetables. Finally, the dietary proteins were chosen so that they showed a high BCAA/AAA ratio, yielding respectively 20.6% and 6.81% of the diet energy content (Fig. 1A). The resulting CD contained less lipids and more carbohydrates and proteins than the traditional KD, and was instead approaching the macronutrients content of the standard diet (SD 0.1:1 ratio; CD 0.6:1 ratio; KD: 6.3:1 ratio; see Fig. 1B for % of caloric supply).

We ascertained whether the different dietary treatments tested in this study were consumed by mice in adequate amount so that it would not alter their body weight. Six weeks old mice fed with SD, CD or KD *ad libitum* showed no change in body mass assessed every third day upon 3 weeks of diets (SD mice: +0.75 ± 0.12 g, n = 6; CD mice: +0.62 ± 0.06 g, n = 5; KD mice: +0.08 ± 0.08 g, n = 6; F < 1; Fig. 1C), suggesting that mice self-regulate their food intake according to the diet (i.e. decrease intake with higher caloric supplies) and adjust their metabolism to keep their body weight constant.

### Effect of diets on excitatory neurotransmission

**Figure 2:**
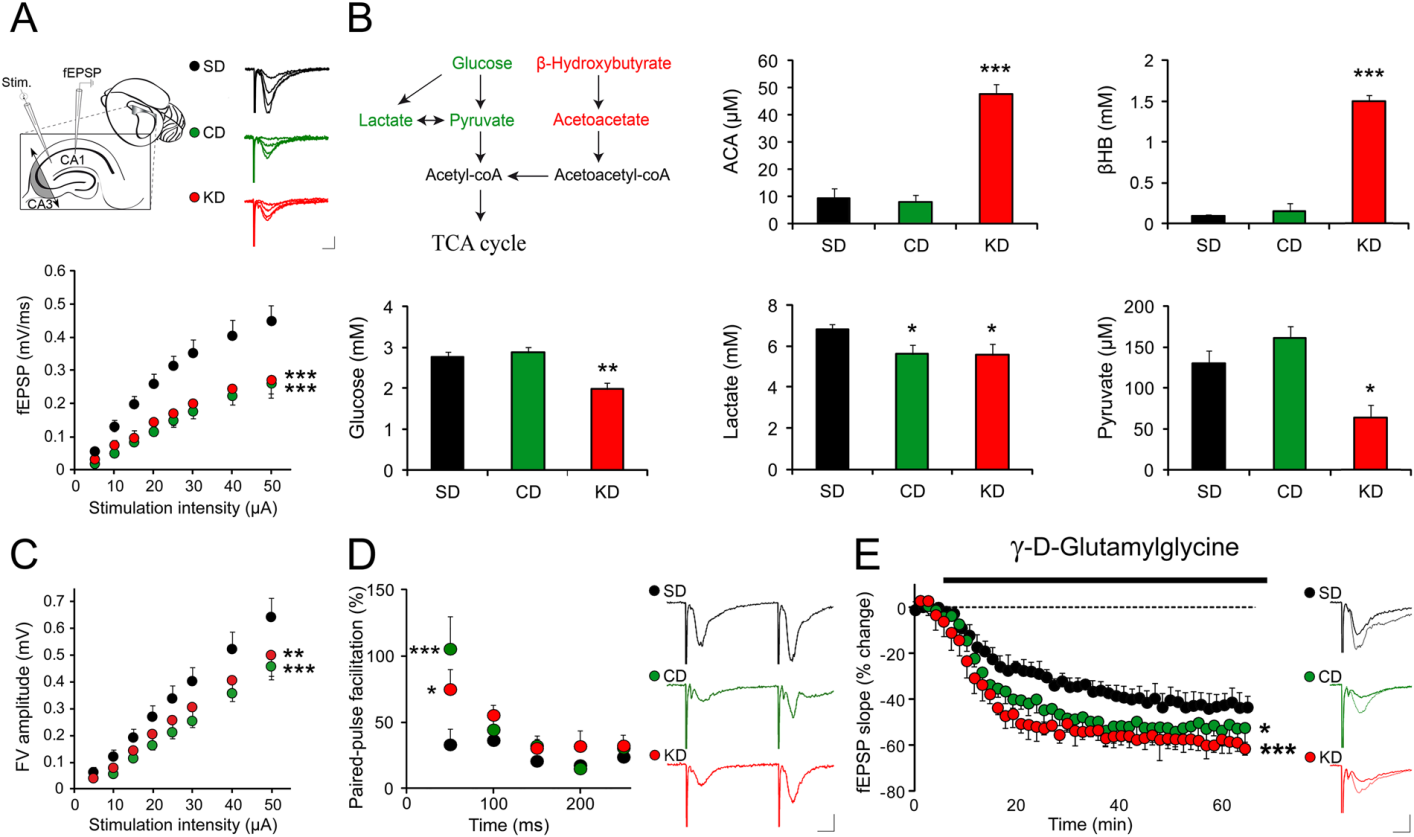
CD treatment reduces excitatory synaptic transmission. **(A**) Hippocampal slices prepared from mice fed with KD or CD displayed reduced basal synaptic transmission compared to control SD fed animals (SD controls, n = 14; CD, n = 7; KD, n = 6; p < 0.001). Calibration bars: 10 ms, 0.5 mV. (**B**) Analysis of blood ketone bodies concentrations revealed a significant increase in both β-hydroxybutyrate (βHB; p < 0.001) and acetoacetate (ACA; p < 0.001) in the KD group only (n = 10). Markers of glycolysis (glucose, p < 0.01; lactate, p < 0.05; pyruvate, p < 0.05) were also significantly reduced in mice supplied with KD, indicating that this diet only induces high level of ketosis. The only change triggered by CD was a significant reduction in lactate (p < 0.05). (**C**) Analysis of the fibre volley amplitude as a function of stimulation intensity revealed a significant reduction in presynaptic excitability of CD and KD fed mice as compared to controls fed SD (CD: p < 0.001, KD: p < 0.01). (**D)** CD and KD dietary treatment also resulted in a clear enhancement of paired-pulse facilitation (SD, n = 5; KD, n = 6, p < 0.05; CD, n = 7, p < 0.001), which indicates a decreased probability of release. Example traces show facilitation at 50 ms ISI. Calibration bars: 10 ms, 0.5 mV. (**E)** The low-affinity competitive antagonist γ-d-glutamylglycine (γ-DGG), at a non-saturating concentration (0.5 mM) at which its potency depends on glutamate concentration decreased neurotransmission to a greater extent in CD and KD fed mice compared to SD controls (SD, n = 9; KD, n = 6, p < 0.001; CD, n = 7, p < 0.05), indicating lower glutamate synaptic concentrations in the KD and CD groups. Calibration bars: 10 ms, 0.5 mV. *p < 0.05, **p < 0.01, ***p < 0.001.

To get insight on whether the CD influences neuronal activity, we first investigated its impact on synaptic transmission. To do so, we assessed excitatory synaptic function at CA3 to CA1 hippocampal synapses using extracellular field potential recordings in acute brain slices from mice fed 3 weeks with SD, CD or KD. Strikingly, input/output relationships revealed that both CD and KD induced a marked reduction in basal synaptic efficacy (Fig. 2A; SD: n = 14; CD: n = 7 KD: n = 6; F_2,192_ = 12.04, p < 0.001 for both diets). To investigate whether such downregulation could be due to high level of ketone bodies, blood samples from mice fed with the different diets were tested for markers of ketosis β-hydroxybutyrate (βHB) and acetoacetate (ACA), as well as markers of glycolysis - glucose, lactate and pyruvate (Fig. 2B). Expectedly, KD fed mice (n = 10) were ketotic, as levels of ketone bodies (βHB and ACA) were markedly increased compared to SD fed mice (n = 16; F_2,23_ = 51.28, p < 0.001), while markers of glycolysis metabolism, i.e. glucose (F_2,29_ = 8.03, p < 0.01), lactate (F_2,29_ = 6.21, p < 0.05) and pyruvate (F_2,29_ = 6.27, p < 0.05), significantly decreased (Fig. 2B). In contrast, CD fed mice blood (n = 6) showed high glucose and pyruvate levels, and very low levels of βHB and ACA, similar to SD controls (all p > 0.05). Interestingly though, lactate concentration was significantly decreased (p < 0.05). These data show that CD, unlike KD, does not favour ketosis, indicating that the down-regulatory effect of the CD on excitatory transmission does not depend on ketone bodies.

We then assessed whether the reduction of synaptic transmission could be attributable to a change in presynaptic excitability, as found in ketotic and non-ketotic calorie restricted rats^24^. To this end, we measured the fibre volley amplitude as a function of stimulation intensity in the CA1 *stratum radiatum* region of the hippocampus. This analysis revealed a significant reduction in presynaptic excitability of CD and KD fed mice as compared to controls fed with SD (Fig. 2C; F_2,192_ = 12.04; CD: p < 0.001, KD: p < 0.01). In order to ascertain that this change in presynaptic excitability influences presynaptic release of neurotransmitters, we assessed paired pulse facilitation, a form of short-term plasticity sensitive to changes in release probability^25^. Paired-pulse profiles of KD and CD fed mice were significantly different from controls (F_2,75_ = 4.52, p < 0.05), with a higher facilitation at the shortest inter-stimulation interval (Fig. 2D; SD: +33.33 ± 11.35%, n = 5; CD: +105.62 ± 25.56%, n = 7, p < 0.001; KD: +75.15 ± 15.14%, n = 6; p < 0.05), indeed indicating a decreased probability of release.

Finally, to investigate whether the decreased presynaptic function resulted in reduced glutamate levels at the synapse we used γ-d-glutamylglycine (γ-DGG), a low-affinity competitive antagonist of the AMPARs, at a non-saturating concentration (0.5 mM) at which its potency depends on glutamate level^26^. γ-DGG inhibition of evoked excitatory fEPSP was significantly stronger in CD and KD slices compared to controls (Fig. 2E; F_2,19_ = 4.76; SD: −40.8 ± 5.0%, n = 9; CD: −51.9 ± 6.6%, n = 7, p < 0.05; KD: −57.4 ± 3.9%, n = 6, p < 0.001), thus indicating lower synaptic glutamate levels in the CA1 hippocampal area of mice fed with CD and KD.

Altogether, these data indicate that CD reduces neuronal excitability and glutamate release at excitatory synapses, as does KD, thus suggesting that a combined non-ketogenic dietary treatment may be an alternative protective strategy against epileptic seizures.

### Susceptibility to acute seizures

**Figure 3:**
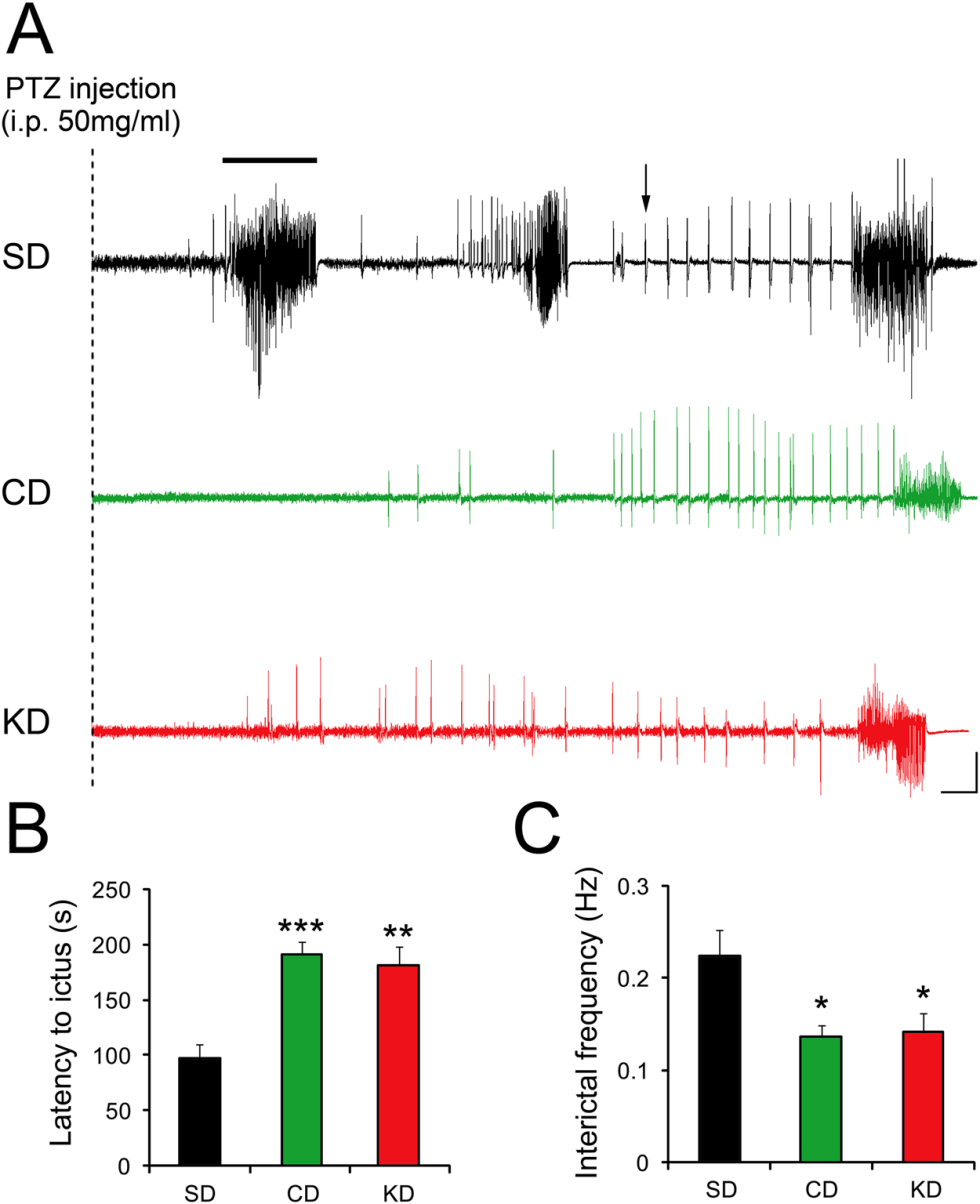
CD treatment decreases susceptibility to seizures. **(A**,**B**) Injection of pentylenetetrazol (PTZ; 50 mg/ml) i.p. promptly resulted in paroxysmal events and status epilepticus in control SD fed mice. In mice receiving KD or CD treatment, the ictus (example indicated by a black bar) was triggered significantly later (SD, n = 7; KD, n = 5, p < 0.01; CD, n = 8, p < 0.001), indicating a similar protective effect of both diets. (**A,C**) Corroborating this finding, the frequency of interictal events (example shown by an arrow) was found to be significantly reduced in both KD and CD fed mice compared to SD controls (all p < 0.05). Calibration bars: 10 s, 500 µV. *p < 0.05, **p < 0.01, ***p < 0.001.

To investigate whether CD could indeed protect against seizures, we first acutely induced paroxysmal events and convulsions by injecting the proconvulsant GABA_A_-receptor antagonist pentylenetetrazol (PTZ, Fig. 3A; 50 mg/ml i.p.) while recording EEG and behaviour using video and telemetric monitoring. No spontaneous seizures or early signs of abnormal activity, such as spike-and-waves could be detected before PTZ injection in mice fed with CD (n = 8), KD (n = 5) or SD (n = 7). The latency to ictus after PTZ injection was 97.14 ± 13.10 s in 9 weeks old male mice fed with SD (Fig. 3B). Strikingly, this latency almost doubled in mice fed with CD (F_2,17_ = 15.48; 191.20 ± 11.08 s, p < 0.001) or KD (180.80 ± 17.00 s, p < 0.01), indicating that mice fed with these diets were less prone to trigger paroxysmal events. Furthermore, the frequency of interictal events was also found to be reduced by ~30% in the CD and KD groups compared to controls (Fig. 3C, F_2,17_ = 5.74; SD: 0.22 ± 0.03 Hz; CD:0.14 ± 0.01 Hz, p < 0.05; KD: 0.14 ± 0.02 Hz, p < 0.05). However, the severity of PTZ-induced convulsive behaviour was comparable between the groups as most paroxysmal episodes were scored ≥ grade 4 in SD (100%), CD (~90%) and KD (100%) groups. Together, these data show that both CD and KD decrease latency to paroxysmal events, but not severity.

### Protection against chronic seizures

**Figure 4:**
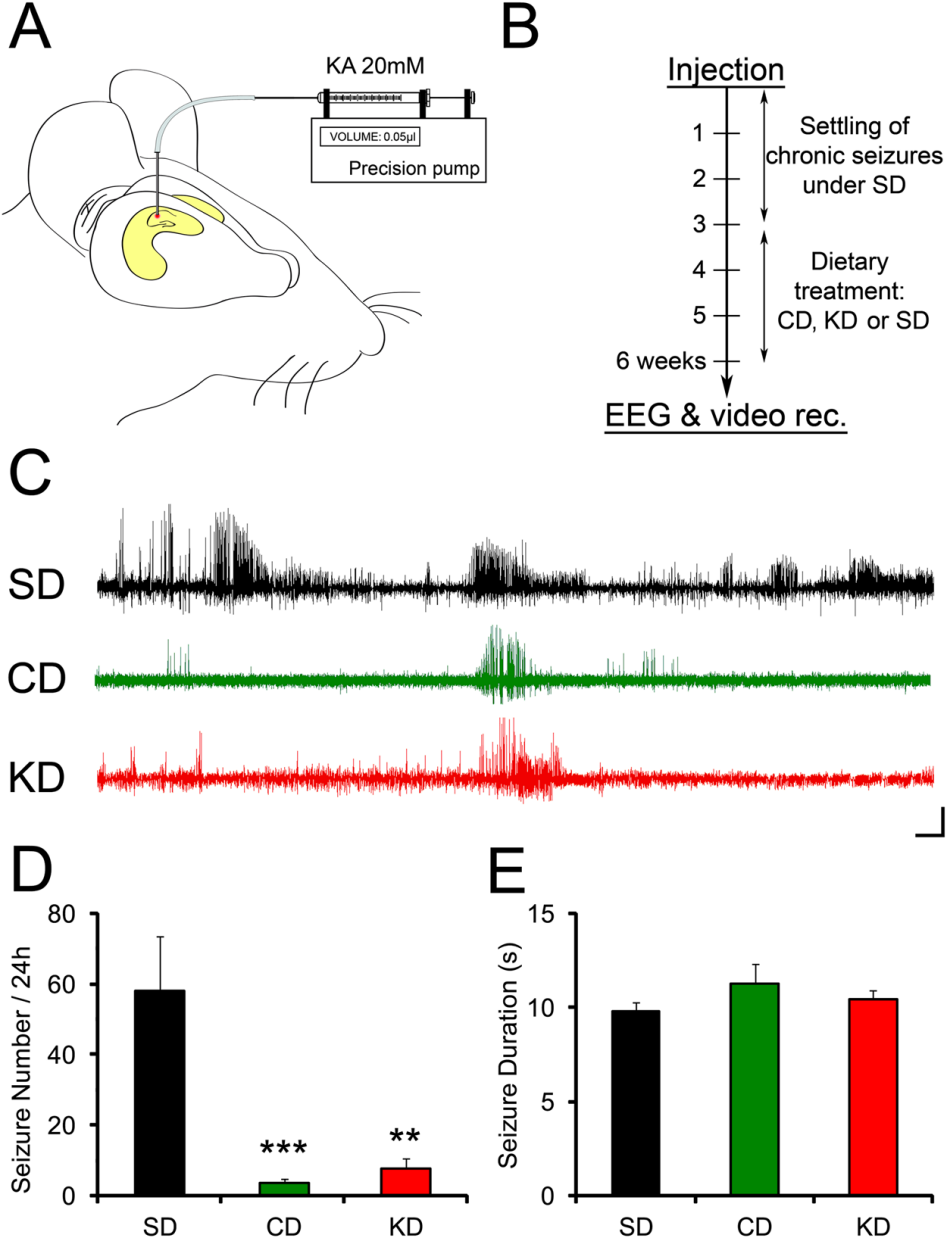
CD treatment reduces chronic seizure occurrence. (**A**) The KA mouse model of chronic epilepsy. KA (20 mM) was injected unilaterally by mean of a cannula connected to a precision pump into the intra-hippocampal region. (**B**) Mice under SD were left 3 weeks to develop chronic seizures and were then fed with KD or CD, controls were left on SD. (**C,D**) EEG detected seizures were drastically decreased in mice treated with KD (n = 8, p < 0.01) and CD (n = 13, p < 0.001) compared with SD controls (n = 10). (**C,E**) EEG detected seizures showed similar durations between SD, KD and CD mice. Calibration bars: 10 min, 200 µV. **p < 0.01, ***p < 0.001.

Induction of seizures with PTZ is an acute *in vivo* assay enabling evaluation of the sensitivity of mice to proepileptic agents. However, it is not a model of chronic epilepsy, which most closely resembles the human epileptic condition. One experimental model of chronic epilepsy is KA-induced temporal lobe epilepsy obtained by performing a unilateral intrahippocampal injection of KA (Fig. 4A). Following a latent period of about 2 weeks, mice develop focal, recurrent seizures interspaced with interictal spikes-and-waves and this lasts for the rest of the animal’s lifespan^27^.

To investigate whether our combined dietary strategy could alleviate chronic epilepsy, we injected KA (20 mM, 0.05 µl) into the CA1 area of the hippocampus of 6 weeks old C57BL/6j males (Fig. 4A), and then waited for 3 weeks for the epileptic phenotype to develop before feeding the mice with SD, CD or KD for an additional 3 weeks period (Fig. 4B). CD fed mice, as KD mice, did not show spontaneous seizures or early signs of abnormal activity without KA injection (n = 4 for both diets; Supplementary Figure 1). The mortality rate of SD fed mice injected with kainate was 10%. No mice fed CD or KD died. Remarkably, while the KA injected SD control group experienced ~60 EEG detected seizures per day (n = 10), there was a protection against development of frequent seizures in both CD (n = 13) and KD (n = 8) fed groups as shown by the drastic difference of ~90% in seizure frequency (Fig. 4C,D; F_2,28_ = 11.98; SD: 57.98 ± 15.39 seizures/day; CD: 3.58 ± 1.24 seizures/day, p < 0.001; KD: 7.71 ± 2.95 seizures/day, p < 0.001). Respectively ~38% (5 out of 13) and ~12% (1 out of 8) of the CD and KD fed mice even showed no EEG detected seizures in the recorded period while 100% of SD fed mice displayed epileptic events (10 out of 10). However, the mean seizure duration was similar for all three groups (Fig. 4D,E; SD: 9.82 ± 0.45 s; CD: 11.25 ± 1.08; KD: 10.43 ± 0.51 s; F < 1).

We then tested whether this protective effect of the CD could be due to a diet-induced reduction in excitability in the epileptic hippocampus. To assess presynaptic excitability in epileptic KA mice, we measured the fibre volley amplitude as a function of stimulation intensity. This analysis revealed a significant reduction in presynaptic excitability of CD and KD fed mice as compared to controls fed with SD (Supplementary Figure 2; n = 7 in each group, F_2, 144_ = 71.409, p < 0.001 for both diets).

**Figure 5:**
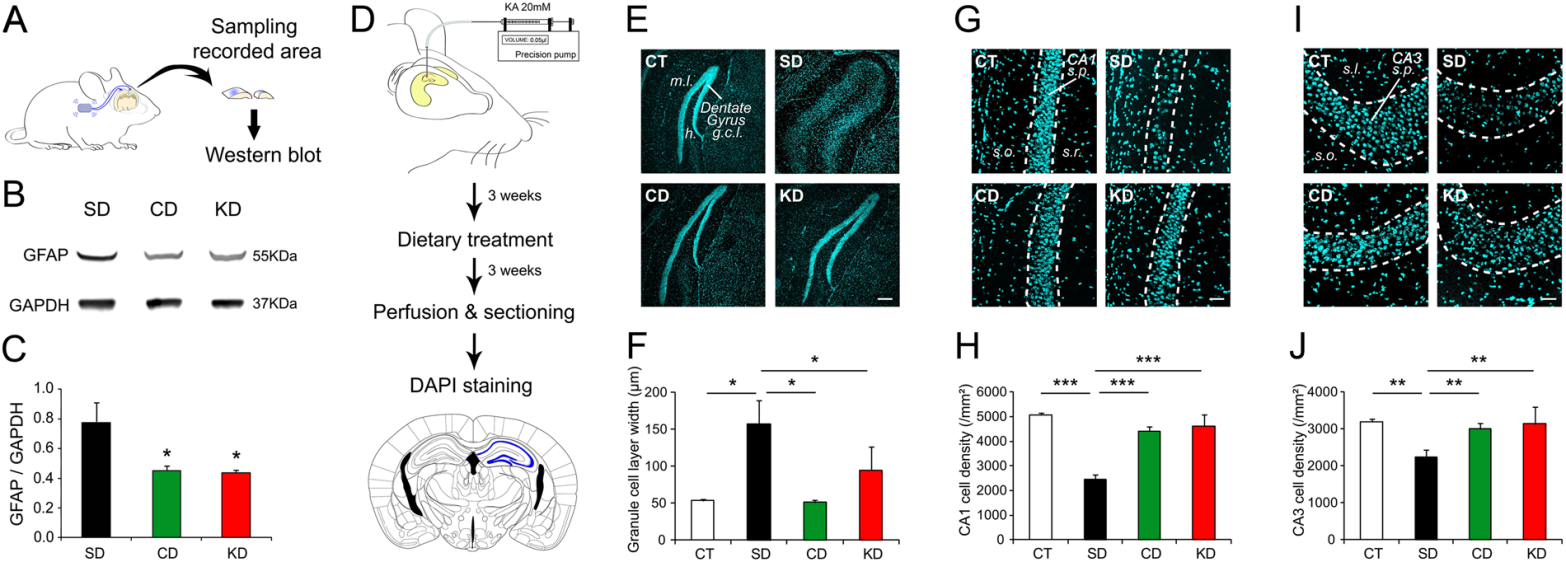
CD treatment rescues cytological and molecular correlates of chronic epilepsy. (**A**) Tissue obtained from the recording site of KA mice was collected for immunoblotting. **(B,C**) Expression of GFAP was significantly reduced in mice treated with KD and CD (n = 4 per group, all p < 0.05). (**D)** KA mice were prepared for histological analysis of hippocampal area CA1, CA3 and DG. This article was published in *The mouse brain in stereotaxic coordinates*, Paxinos, G & Franklin, K, p.98, Copyright Elsevier Academic Press, 2001. This figure is not covered by the CC BY licence. Elsevier. All rights reserved, used with permission. (**E,F)** Both CD and KD (CD, n = 5; KD, n = 5) fed mice showed protection against dispersion of the granule cell layer (g.c.l.) compared to SD fed mice (SD, n = 6) and mice that did not receive KA (CT, n = 3).*p < 0.05. (**G**–**J)** CD and KD fed mice were also protected against cell loss in CA1 and CA3 compared to SD animals. **p < 0.01, ***p < 0.001. (**h**) hilus, m.l.: molecular layer, s.l.: stratum lucidum, s.o.: stratum oriens, s.p.: stratum pyramidale, s.r.: stratum radiatum.

Gliosis is a typical hallmark in chronic temporal lobe epilepsy and may contribute to the disease phenotype. Molecular correlates of chronic epilepsy were assessed by examining the levels of glial fibrillary acidic protein (GFAP) in the cortical regions from which electrophysiological recordings were obtained (Fig. 5A). Consistent with the marked reduction in EEG detected seizure frequency, CD and KD fed mice also showed significantly less GFAP immunoreactivity than controls, as assessed by western blotting (Fig. 5B,C; n = 4 per group; F_2,9_ = 5.59, p < 0.05 for both treatment).

Finally, neuroanatomical correlates of recurrent seizures were also considered by assessing dispersion of the granule cell molecular layer of the dentate gyrus, as well as cell loss in the hippocampal CA1 and CA3 areas, typical consequences of chronic temporal lobe epilepsy (Fig. 5D). SD fed mice classically displayed a large dispersion of the granule cell layer as compared to control mice that did not receive KA injection. Strikingly, there was no dispersion in CD fed mice and dispersion was significantly reduced in KD fed animals (Fig. 5E,F; F_3,15_ = 5.26, p < 0.05 for both treatments). Cell loss in CA1 and CA3 hippocampal areas was also limited in both CD and KD groups (Fig. 5G–J; CA1: F_3,15_ = 17.19, p < 0.001 for both treatments; CA3: F_3,15_ = 8.42, p < 0.01 for both treatments).

As a whole, our study indicates that the CD strategy decreases excitatory drive, lessen the propensity to paroxysmal events, and reduces anatomical and histological markers of epileptogenicity.

## Discussion

The current study aimed at ascertaining whether preferential intake of specific nutrients could downregulate excitatory neurotransmission and yield protection against seizures. To our knowledge, this is the first attempt to assess the effect of combining several antiepileptic nutritional strategies aiming at dampening excitatory transmission and seizure occurrence. Evaluation of synaptic function at Schaffer-collaterals CA3 to CA1 hippocampal synapses indicated that mice fed with this combined diet indeed show significantly reduced excitatory neurotransmission attributable to a decreased presynaptic excitability and hence, a reduced probability of glutamate release. As well, CD fed mice are much less susceptible to PTZ induced seizures and are largely protected against chronic seizures as well as cytological and molecular changes in the KA model of epilepsy.

Most interestingly, unlike KD fed mice, animals under CD did not enter ketosis and still displayed comparable changes in neurotransmission and susceptibility to seizures. These results support the view that ketone bodies do not fully explain the decrease in excitation associated with the classical KD. A lack of correlation between ketone bodies concentration and protection against seizures has indeed been reported (e.g. refs 16 and 28). This finding further corroborates *in vitro* studies showing no inherent effect of βHB or ACA on synaptic transmission^29, 30^, although an acute application of these compounds overlooks the chronic effects likely occurring in these protracted treatments. Kim and collaborators indeed showed that chronic administration of ketone bodies is sufficient to reduce seizures in the kcna1-null mouse model of epilepsy^31^. Interestingly, most investigations that examined the intrinsic effect of ketone bodies by direct application in glucose containing artificial cerebrospinal fluid (ACSF) found no effect on synaptic function^29, 30^ while replacement of extracellular glucose with βHB or ACA reduces neurotransmission^32^. Therefore, a conspicuous yet recurrent question in KD research is whether the reduction in excitation is attributable to lipid metabolism or to dormant glucose metabolism. The latter possibility is supported by several investigations showing that glycolytic inhibition alone does reduce epileptic events in rodent models of epilepsy^17, 18, 33^. Moreover, a recent study provides some ground for these effects by demonstrating that blocking the glycolysis metabolic enzyme lactate dehydrogenase (LDH) is sufficient to hyperpolarize neurones and protect against seizures^4^. Our findings are also in line with this, as mice maintained on CD showed no increase in ketone blood content and nonetheless showed a similar decrease in neurotransmission as KD fed mice. The decrease in lactate observed in both CD and KD groups also favours this interpretation as it may be due to lower LDH activity in converting glucose and pyruvate to lactate. This is indeed a possible consequence of a low glycaemic index diet^34^, enriched BCAA^35^, as well as high levels of medium-chain triglycerides^36^ and polyunsaturated fatty acids^37^. Importantly, although synaptic glutamate concentrations have previously been proposed to be reduced in KD^23^, we here further suggest that such reduction is, at least in part, due to a decreased presynaptic excitability leading to less glutamate release. These findings are in line with previous *in vivo* investigations reporting a decreased hippocampal excitability in calorie-restricted and KD fed rodents^24, 38^. Interestingly, the reduction in fibre volley we report in CD and KD fed epileptic mice is more profound than in control CD and KD fed mice. This differential effect might be explained by cellular alterations and homeostatic changes that occur following kainate injection even when cell death is not prominent. Such effect may not be seen in control SD fed epileptic mice as the increase in presynaptic excitability would increase the fibre volley while neurodegeneration would decrease it, eventually resulting in no change. Insofar as both CD and KD induce similar changes in neurotransmission, and that these are consistent with the neuronal hyperpolarization showed by Sada *et al*. (2015)^4^, it is indeed likely that the nutritional strategies employed in our CD concur to downregulate the LDH metabolic pathway, a component of the astrocyte-neurone lactate shuttle. It also remains probable that other mechanisms act in parallel to dampen neuronal excitation, such as for instance reduced glutamate level in the brain that could result from a high BCAA/AAA ratio^19, 20^, or neuronal hyperpolarization induced by polyunsaturated fatty acids though modulation of voltage gated ion channels^39^, or the increase in adenosine receptors activation under medium-chain triglycerides treatment, which reduces probability of neurotransmitter release^40^.

Although the classical KD is less deleterious in terms of general health than one might expect in regard to its high fat content, various side effects of the diet have been reported. These include defects in growth (in children), lipid levels, cardiac function, kidney stones, haematology, immunology, metabolism (acidosis, hyperuricemia), as well as gastric issues such as constipation, diarrhoea, nausea and vomiting^41^. The most common complication of KD is hyperlipidaemia, with increased levels of triglycerides, apolipoproteins and very low and low density lipoproteins (VLDL and LDL)^42^. This side effect is however reversible upon interruption of the KD. In this regard, the combined nutritional strategy we propose herein would most likely be beneficial as, besides its much lower fat content, it provides several nutrients with beneficial effects. Indeed, polyunsaturated fatty acids provided in rapeseed oil were found to improve lipid content of children with hypercholesterolaemia^43^ and a polyunsaturated fatty acids rich olive oil based KD showed reduced dyslipidaemia^44^. Medium-chain fatty acids have also been found to reduce cholesterol, triglycerides and lipoproteins levels^36^. Interestingly, several studies also suggest that preferential intake of low-glycaemic index carbohydrates reduces lipid blood content^45, 46^. Growth retardation is also a detrimental side effect often observed in children on KD. It has been suggested that the risk of growth impairment might be lower on diets allowing higher protein consumption and decreased fat intake^47^. Although no study is available to confirm this hypothesis, if it held true, the more balanced combined strategy used in our study might avert concerns about growth.

The unpalatability leading to difficult adherence mostly explains why KD is less commonly used in adult treatments^48^. To circumvent this issue, Kossoff *et al*. developed a less restrictive protein rich KD called “modified Atkins diet” (MAD) with the aim of ameliorating diet palatability. Such strategy proved to be efficient as approximately half of the patients displayed reduction in seizure frequency >50%^49^. Yet, although the diet is well tolerated among patients, hyperlipidaemia is still commonly observed^50^. The nutritional strategy proposed herein may be more palatable than the traditional KD, as it comprises a higher proportion of carbohydrates (~15% *vs*. ~10% in the MAD) and similar proportions of proteins and fat. Given the beneficial impact of polyunsaturated fatty acids and low glycaemic index^46^ on health parameters including blood lipid content, and the manageability of medium-chain triglycerides gastrointestinal side effects, it is also reasonable to anticipate that the CD approach may be well tolerated by patients.

Other positive features of the proposed dietary strategy stem from recent investigations showing that medium-chain triglycerides and polyunsaturated fatty acids have a beneficial impact on cognition, mostly by slowing down its decline in pathology or ageing^51, 52^. Given that epileptic conditions are often deleterious to cognitive functions, besides its protection against seizures, the CD approach may also offer appreciable procognitive effects. This assumption is supported by our finding that CD decreases presynaptic excitability. Indeed, although the well accepted synaptic plasticity and memory hypothesis suggests that higher neuronal excitability would favour the long-lasting changes in the strength of synaptic connections thought to underlie memory formation^53^, several investigations suggest that decreasing neuronal excitability would actually be beneficial to cognition as it enhances the dynamic range for plasticity and reduces the signal-to-noise ratio. Perhaps the most direct evidence of this effect is the enhancement of cognitive functions in both humans and rats by cathodal transcranial direct current stimulations, known to decrease excitability by hyperpolarizing neurons^54, 55^. Similarly, caloric restriction, which decreases excitability^24, 38^, has been found to improve cognitive performances in humans and mice^56, 57^. Finally, BCAA have been found to help cognitive recovery following traumatic brain injury^58^ and may therefore show similar recuperation properties on seizure-induced lesions.

In summary, by comparing the effect of the KD with the combination of several dietary antiepileptic strategies on basal excitatory neurotransmission and their consequences in terms of susceptibility to acute or chronic seizures, our study provides the proof of concept that the association of specific nutrients into the same non-ketogenic regimen confers robust protection against seizures. Such diet is more balanced (lower [fat]:[proteins + carbohydrates] ratio) than the classic KD or MAD and contains nutrients providing several beneficial impacts on general health, including cardiovascular health and cognitive functions. Although side-effects and toxicology tests are required, it would now be of prime interest to perform clinical trials using a similar combined approach.

## Methods

### Animals

Experiments were carried out according to the guidelines of European Community Council Directives of 01/01/2013 (2010/63/EU) and our local animal care committee (Center for Interdisciplinary Research in Biology in College de France). Experiments were performed on 6 to 12 weeks old wildtype C57BL/6j male mice. All efforts were made to minimize the number of used animals and their suffering.

### Ethical and accordance statements

Experiments were approved and carried out in accordance with the national ethical committee for animal experimentation number 59, authorization number 2015071010466740.

### Diets

Mice were fed with standard diet (SD), CD, or KD. SD was provided by the Teklad 2018 diet. KD was provided by the rodent Bioserv F3666 specific diet. PILEJE Laboratoire provided the CD.

### Biochemical analyses of ketosis and glycolysis markers

Levels of glucose, lactate, pyruvate, β-hydroxybutyrate, and acetoacetate were measured on whole blood (500 µl drawn by cardiac puncture after pentobarbital anaesthesia of the animal) deproteinized (with one volume of perchloric acid) using spectrophotometric enzymatic methods as described in ref. 59.

### *Ex vivo* electrophysiology

Acute transverse hippocampal slices (400 μm) were prepared as previously described^60, 61^. Briefly, mice were sacrificed by cervical dislocation and decapitation. The brain was rapidly removed and placed in chilled (1–4 °C) ACSF composed of (in mM): 119 NaCl, 2.5 KCl, 2.5 CaCl_2_, 1.3 MgSO_4_, 1 NaH_2_PO_4_, 26.2 NaHCO_3_, 4 glucose, 7 sucrose). Hindbrain and forebrain were excised and the brain laid flat on the rostral aspect. Fine curved blunt forceps were used to remove the midbrain and the majority of the white matter. The brain was then laid dorsally and the hippocampi dissected out. Slices were maintained at room temperature in a storage chamber containing ACSF saturated with 95% O_2_ and 5% CO_2_ for at least 1 h before the experiments.

Slices were then transferred to a submerged recording chamber mounted on a Scientifica SliceScope Pro 6000 microscope equipped for infrared-differential interference (IR-DIC) microscopy and were perfused with ACSF (2 ml/min). All experiments were performed in CA1 *stratum radiatum* region of the hippocampus. Field excitatory postsynaptic potentials (fEPSPs) were recorded in the presence of the GABA-receptor inhibitor picrotoxin (100 µM) with glass pipettes (2–5 MΩ) filled with 1 M NaCl. A cut was made between CA1 and CA3 to prevent the propagation of epileptiform activity (Fig. 2A). Postsynaptic responses were evoked by stimulating Schaffer collaterals (0.033 Hz) in CA1 stratum radiatum with ACSF filled glass pipettes. Input/output relationships of evoked excitatory postsynaptic potentials (EPSPs) were assayed by incrementing stimulation strength (5 to 50 µA, 100 µs). The test-shock used in subsequent experiments was chosen to elicit 50% of the maximal slope. Paired-pulse experiments consisted of 2 identical stimuli with increasing interpulse intervals (50 to 250 ms). Paired-pulse ratios were generated by plotting the maximum slope of the second fEPSP as a percentage of the first.

### Telemetric EEG and video recording

EEG experiments were carried out using wireless ETA-F10 transmitters (Data Sciences International) for chronic EEG recording and video monitoring. After anaesthesia (ketamin, 95 mg/kg; xylazin, 10 mg/kg; intraperitoneal), a 1 cm midline sagittal incision was made starting above the skull midline and extending along the neck to create a pocket for subcutaneous placement of the transmitter along the dorsal flank of the animal. The flexible recording electrodes were implanted subdurally through small holes drilled in the skull (stereotaxic coordinates: −2 mm AP, +1.5 and −1.5 mm lateral) and held in place with dental cement. Mice were allowed to recover for 7 days before recording.

EEG signal was collected through DSI radiofrequency receivers placed under each cage. EEG data were acquired at a sampling rate of 200 Hz using the DSI Dataquest A.R.T. system, version 4.33.

### PTZ seizure model

Nine weeks old mice implanted with wireless ETA-F10 DSI EEG electrodes were injected i.p. with 50 mg/kg of PTZ and placed in an open arena for video and EEG recording of the *status epilepticus*. Protective properties of diets were assessed by feeding the mice with KD, CD or SD (controls) for 3 weeks before the PTZ experiment, scoring *status epilepticus* grades according to Ono *et al*.^62^ and evaluating the latency to ictus as well as the frequency of interictal events. The Racine’s scale used to *grade status epilepticus* was as follows: 0, behavioural arrest; 1, rhythmic mouth and facial movements; 2, head nodding; 3, forelimb clonus; 4, rearing and bilateral forelimb clonus; 5, rearing and falling or jumping. Interictal events were spikes or spike trains with a minimal amplitude of 1.5X baseline, and a maximal duration of 5 seconds. The number of detected interictal events was then counted manually.

### KA mouse model of chronic epilepsy

Six weeks old C57BL/6j mice were injected unilaterally with kainate (0.05 µl, 20 mM in saline) at a rate of 0.05 µl/min in the CA1 area of the right hippocampus (−2 mm antero-posterior, +1.5 mm lateral, −1.2 mm dorso-ventral) by mean of a stereotaxic apparatus and a cannula (0.29 mm diameter) connected to a precision pump. Mice were then left on SD for 3 weeks for settling of the chronic epileptic phenotype. To assess therapeutic potential of the diets, KA mice were then fed for 3 weeks with KD, CD or SD (controls) and recorded with ETA-F10 DSI EEG electrodes for 72 h in their home cage. Analysis of electrographic seizures, i.e. seizures detected on the EEG recording, was performed using NeuroScore software, and defined as spike trains with a minimal amplitude of 1.5X baseline, and a minimal duration of 5 seconds at a frequency ≥1 Hz^63, 64^. The number of seizures was averaged per day. Artefacts were automatically detected using a high detection threshold and removed manually.

### Immunoblotting

Cortical tissue surrounding the subdural electrode (Fig. 5A) was sampled and homogenized in 2% SDS with protease inhibitor mixture, β-glycerophosphate (10 mM), and orthovanadate (1 mM). Equal amounts of protein were separated on a 10% PAGE gel followed by transfer to nitrocellulose membranes. Proteins were detected by immunoblotting using the HRP-ECL kit from Perkin-Elmer. GAPDH was used as loading control. Primary antibodies used were: GAPDH rabbit monoclonal antibody (Sigma), GFAP rabbit polyclonal antibody. Donkey anti-rabbit IgG (Amersham Biosciences) HRP-conjugated secondary antibodies were used.

### Brain processing, DAPI staining and analyses

Six weeks after intrahippocampal injection of KA, mice were anesthetized, perfused transcardially with PBS 0.1 M and their brains were rapidly removed and frozen in isopentane cooled at −30 °C. Coronal sections (20 μm) were then cut on a cryostat (Microm Cryo-Star HM 560), collected on slides, and fixed with 4% paraformaldehyde in PBS for 30 min at 4 °C. Slices were then processed for staining with the nucleus marker DAPI, mounted with fluoromount and left overnight to set before imaging with a confocal laser-scanning microscope (Leica TBCS SP2, SP5) with a 10X and 60X objective. The fluorescent images were analysed with ImageJ. CA1 and CA3 areas as well as the dentate gyrus (DG) of the hippocampus were independently outlined, and an intensity threshold was set to identify pixels that were DAPI-positive. Cells were then counted in areas of interest using the ImageJ built-in particle analyser and their density (number of cells per mm²) was determined.

### Statistics

All data are expressed as mean ± SEM and n represents the number of independent experiments. Statistical significance was determined by one-way and two-way ANOVA using Statistica 6.1 (Statsoft Inc.) and Statview 5.0 (SAS Institute Inc., Cary, USA). When relevant, a Newman-Keuls post hoc analysis was performed.

## Electronic supplementary material

Supplementary information
