# Supplementary material for: Non-ketogenic combination of nutritional strategies provides robust protection against seizures
Source: Sci Rep. 2017 Jul 14;7:5496. doi: 10.1038/s41598-017-05542-3 (PMC5511156; doi:10.1038/s41598-017-05542-3)
Supplement: Supplementary file 1 — Supplementary information [file 41598_2017_5542_MOESM1_ESM.pdf]

Supplementary information

Non-ketogenic combination of nutritional strategies allows robust protection against seizures

Glenn Dallérac[1]*, Julien Moulard[1], Jean-François Benoist[2], Stefan Rouach[1], Stéphane Auvin[2], Angèle Guilbot[3], Loïc Lenoir[3], Nathalie Rouach[1]

[1] *Neuroglial Interactions in Cerebral Physiopathology, Center for Interdisciplinary Research in Biology, Collège de France, CNRS UMR 7241, INSERM U1050, Labex Memolife, PSL Research University, Paris, 75005, France.*

[2] *AP-HP, Hôpital Robert Debré, Service de Neurologie Pédiatrique , Paris , France.*

[3] *Laboratoire PILEJE, 37 quai de Grenelle, 75015 Paris, France.*

Supplementary information

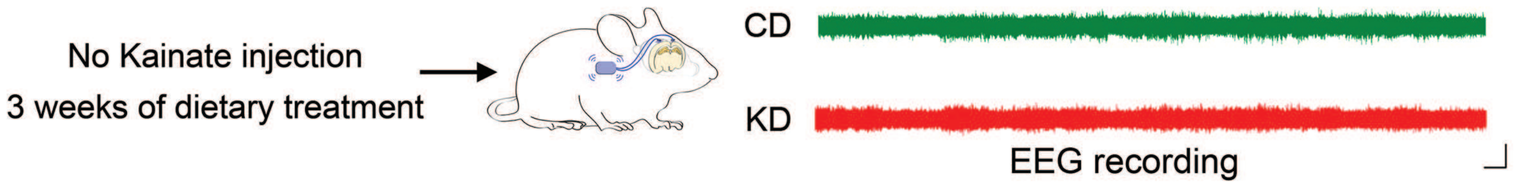

Supplementary Figure 1: CD and KD diets do not induce epileptiform activities. Mice fed for 3 weeks with CD (n=4) or KD (n=4) did not show any EEG-detected seizures. Traces show examples of 4 hours baseline EEG recordings. Calibrations: 10min, 100μV.

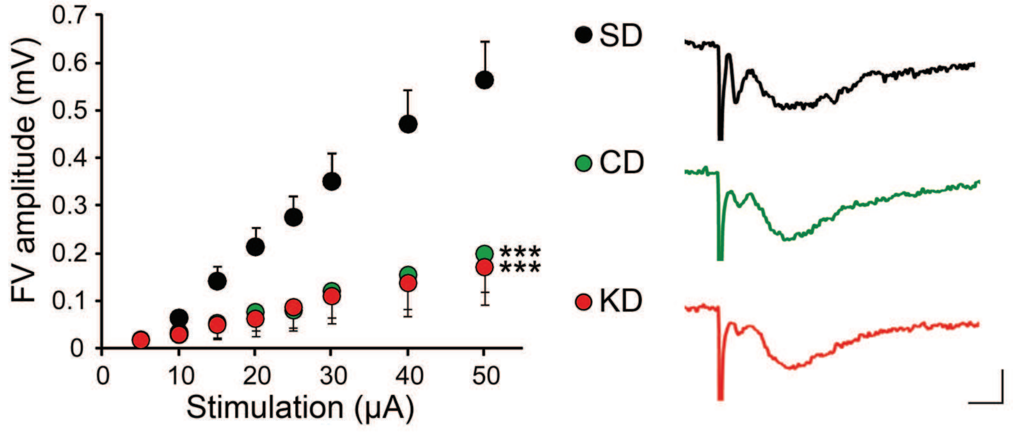

Supplementary Figure 2: CD and KD diets induce a reduction in excitability in the epileptic hippocampus. Analysis of the fibre volley amplitude as a function of stimulation intensity revealed a significant reduction in presynaptic excitability of CD and KD fed mice as compared to controls fed with SD (n=7 in each group; p<0.001 for both CD and KD). Calibrations bars: 5ms, 0, 2mV. *p<0.001.
